# Be aware of the sodium intake outside student canteens: development and validation of a sodium food frequency questionnaire in Chinese undergraduates

**DOI:** 10.3389/fnut.2023.1062845

**Published:** 2023-06-08

**Authors:** Yue Xi, Caihong Xiang, Jiajing Liang, Jiaqi Huo, Cuiting Yong, Hanshuang Zou, Yunfeng Pan, Minchan Wu, Qingqing Xie, Jing Deng, Lina Yang, Jihua Chen, Yufei Qi, Ying Li, Qian Lin

**Affiliations:** ^1^Department of Epidemiology, School of Public Health, Sun Yat-sen University, Guangzhou, China; ^2^Department of Nutrition Science and Food Hygiene, Xiangya School of Public Health, Central South University, Changsha, China; ^3^Department of Preventive Medicine and Hospital-acquired Infection Control, Shenzhen People’s Hospital, Shenzhen, China; ^4^The Biobank of Xiangya Hospital, Central South University, Changsha, China; ^5^Department of Epidemiology and Health Statistics, Xiangya School of Public Health, Central South University, Changsha, China; ^6^Hunan Provincial Key Laboratory of Clinical Epidemiology, Changsha, China; ^7^Department of Physical Education and Research, Central South University, Changsha, China; ^8^Department of Health Management, The Third Xiangya Hospital, Central South University, Changsha, China; ^9^Hunan Key Laboratory for Bioanalysis of Complex Matrix Samples, Changsha, China

**Keywords:** food frequency questionnaire, sodium, validity, college students, salt reduction

## Abstract

**Background:**

Chinese college students used to eat in student canteens, making dietary consumption outside the cafeterias the main reason for the difference in sodium intake. This study aims to develop and validate a food frequency questionnaire (Sodium-FFQ) targeting dietary sodium intake outside the canteens among undergraduates in China.

**Methods:**

This cross-sectional study included 124 and 81 college students from comprehensive universities in the development and validation stage. A 24 h dietary recall and a food frequency questionnaire were used to develop the Sodium-FFQ. Food items were selected according to the foods that contributed more to the total sodium intake. Test–retest correlation coefficients with an interval of 14 days were employed to evaluate reproducibility. Validity was assessed against a single 24 h urine collection and a 3-day dietary record using correlation coefficients, *Bland–Altman* analyses, and cross-classification analysis of *Kappa* coefficients.

**Results:**

The Sodium-FFQ consists of 12 groups of foods with 48 items. The *Spearman* correlation coefficient of test–retest on sodium intake was 0.654 (*p* < 0.05), and that between the Sodium-FFQ, 3 × 24 h dietary record, and 24-h urinary sodium were 0.393 (*p* < 0.05) and 0.342 (*p* < 0.05), respectively. The Sodium-FFQ was correlated to 24 h urinary sodium-to-potassium ratio, with a *Spearman* coefficient of 0.370 (*p* < 0.05). The classification agreement of the Sodium-FFQ and 24 h urinary sodium was 68.4%, and the *Kappa* coefficient was 0.371 (*p* < 0.001).

**Conclusion:**

The Sodium-FFQ developed in this study presented an acceptable reproducibility, validity, and classification agreement. It indicates that the Sodium-FFQ could be a potential tool for promoting sodium restriction in college students.

## 1. Introduction

Excess dietary sodium intake is associated with high blood pressure (1–3), high risk of cardiovascular disease, all-cause mortality, and other chronic diseases (1, 2). Salt reduction programs, regarded as one of the most cost-effective strategies (4), have been implemented worldwide with a great diversity of approaches (4–7). However, the problem remains: most people have no idea how much sodium they have taken in (8). Therefore, an assessment tool that monitors sodium intake and identifies the source of sodium is strikingly needed.

Biomarker detection and dietary survey are the main ways of assessing sodium intake (9). Compared with detecting sodium concentration in the urine or blood, the latter is more straightforward, convenient, and widely applied to collect dietary information in epidemiological studies (10). The food frequency questionnaire (FFQ) is a common dietary survey method (10, 11). A well-designed FFQ could better reflect the dietary intake level of certain nutrients and the risk of malnutrition and even be transformed into a practical tool for self-assessment (10, 12, 13). Several studies have evaluated residents’ dietary sodium (or salt) intake by designing a sodium-related FFQ (7, 14–17). In contrast, such a study is scarce (18, 19) in China due to the patterns and typical communal meals.

College students, who are in the emerging period (usually defined as 18–25 years old), are unique and vulnerable (20, 21). Most undergraduates eat in the student canteens or restaurants around the school (including eating take-out foods). Thus, deciding and estimating how much salt and high-sodium seasonings to add and eat is hard. Consequently, the foods outside the canteens, like processed foods, have become the leading cause of dietary sodium differences among this population. College years are vital for transforming and establishing dietary behaviors and habits (20, 21). Forming and keeping a low-sodium diet will help college students’ life-long health. A practical tool would present the daily sodium intake of the undergraduates, act as an alert, and guide them to make better food choices.

Therefore, this study aimed to develop a sodium food frequency questionnaire focused on foods outside student canteens for college students and validate it via testing against 24-h urinary sodium excretion and 3-day dietary records.

## 2. Materials and methods

### 2.1. Ethical approval

The study was approved by the Ethics Review Committee of the Xiangya School of Public Health, Central South University (XYGW-2020-087).

### 2.2. Study design and participants

This is an observational study with a cross-sectional design. The study was conducted from November 2020 to October 2021 in Changsha City, Hunan Province, China. College students studying in the comprehensive universities were recruited according to the following criteria: (1) without severe liver and kidney insufficiency, cardiovascular and cerebrovascular diseases, benign tumors, or mental diseases, (2) with a regular diet in the past month, and (3) ability to read and write. The following exclusion criteria were added for the students required to collect urine: (1) being menstruating, (2) taking diuretics in the last 2 weeks, and (3) being unable to collect 24-h urine. All participants signed the informed consent before the study. A total of 124 and 81 students participated in the development and validation stages, respectively.

### 2.3. Development of The sodium-FFQ

#### 2.3.1. Stage 1

A convenience sample of 151 students from comprehensive universities who met the abovementioned criteria was recruited, and 124 provided complete dietary information. A one-day dietary recall and food frequency questionnaire targeting the past 12 months was employed to identify the college students’ high-sodium foods frequently consumed in winter, spring, and autumn. Food items were first selected from one-day dietary recall data and supplemented by data from an FFQ derived from China Health and Nutrition Survey (CHNS) (22). The contribution rate of each food to the total sodium intake was calculated, and the foods with a contribution rate reaching 90% were selected for the food list. Besides, the foods with a content ≥200 mg Na/100 g in the Chinese Food Composition List and the commercially processed foods (Na ≥ 500 mg Na/100 g) were also added (10, 12, 23). In this stage, a total of 12 categories with 55 items were included in the draft version of the FFQ.

Responses to each item were designed in a fill-in-blank format. The consumption frequency, with the units of ‘time(s)/month,’ ‘time(s)/week,’ and ‘time(s)/day,’ was converted as the daily frequency divided by 28 days a month. Standard portion sizes were determined referring to the most frequent answers from the one-day dietary recall data and varied by different eating ways, such as ‘a spoon/portion from aunts in the canteen’ or ‘a bowl from homemade dishes.’

- The total sodium intake was estimated using the formula below:

$$Total\;Na=\overset{n}{\underset{i=0}{\sum }}F_{i}\times I_{i}\times C_{Na,i}$$

Total sodium intake from FFQ (*Total Na*, mg/d), food items (*i*), frequency (*F*, times/d), intake of each time (*I*, g, or mL), and sodium content of each food (*C*, mg/g or mL/L), and *n* is a natural number.

- Adjusted sodium intake was the total Na adjusted by the items regarding “re-adding salt to the cooked meals” of the subjects, and the model was as follows:

$$\begin{array}{l} AdjustedSodiumIntake\;\left(mg/d\right)=Total\;Na+AS\times F_{AS}\times \\ m_{AS}\times 400 \end{array}$$

Total sodium intake from FFQ (*Total Na*, mg/d), add salt again or not (*AS*, Yes = 1, no = 0), frequency of adding salt (*F_AS_*, occasionally = 1/7 = 0.1429, often = 3/7 = 0.3571), grams of salt added each time (*m_AS_*, one spoonful = 2 g, unclear = 3 g, or the actual amounts added each time), and the conversion factor (1 g salt ≈ 400 mg sodium).

#### 2.3.2. Stage 2

Two experts in nutrition evaluated the representativeness, response format, and standard portion sizes of each item for college students. Then, thirteen foods rich in sodium (4) or potassium (9) were added to the food list; the high-potassium foods were served as the adjustment factor and a reminder of a healthy diet. Here developed the original version of a 68-item Sodium-FFQ.

#### 2.3.3. Stage 3

The original Sodium-FFQ was pre-tested among nineteen volunteer students (five boys and fourteen girls). The comprehensibility of the FFQ was assessed via face-to-face interviews with trained research assistants. Moreover, a single 3-day dietary record and twice food frequency questionnaire were conducted to validate the FFQ preliminarily and confirm the data collection process. After the pre-tested stage, twenty-five items were eliminated, three added, and two modified; the result was the forty-eight-item version of the FFQ, named the 1^st^ version of Sodium-FFQ (Sodium-FFQ 1.0, Supplementary Material).

### 2.4. Validation of the sodium-FFQ

**Figure 1:**
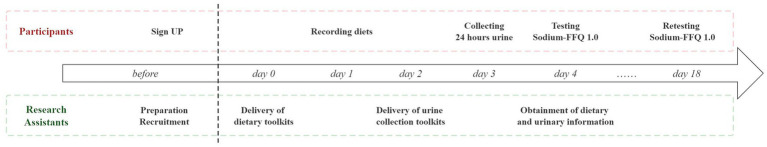
Timeline of validation stage on Sodium-FFQ 1.0.

According to the previous research (24, 25), the sample size was calculated at 100 in this stage (24, 25). Through campus advertisement and online webpage recruitment, one hundred and twenty-four students registered for participation, of which 102 were recruited. In this stage, the online questionnaires were delivered twice, with a 3-day diet record and a single 24 h urine collected then. Figure 1 shows the timeline of this stage.

#### 2.4.1. Test–retest of sodium-FFQ 1.0

The test–retest procedure evaluated the reproducibility over a 14 day interval. Uniformly trained research assistants guided the college students to complete the Sodium-FFQ 1.0 on the 4^th^ and 18^th^ days. The Sodium-FFQ 1.0 was delivered online via The Questionnaire Star, a tool for developing electronic questionnaires. Each questionnaire had a unique linkage. All food items and each type of portion size were displayed with pictures for reference.

#### 2.4.2. Three-day dietary record

Research assistants delivered the standardized dietary record toolkits to the participants and guided them to fill in the record book the day before the start. The toolkit consists of a record book of 3-day diets, an estimated food weight list, a piece of graph paper to examine the amount the participants estimated, and a pen. The participants were asked to choose one of the 3 days to take photos of all the foods on graph paper before and after eating and then send photos to research assistants for examination (26).

#### 2.4.3. Twenty-four-hour urine collection

Since approximately 85 to 90% of sodium is ingested over 24 h of excretion in the urine, the 24 h U_Na_ is considered the gold standard for measuring sodium intake (2, 3). The 24 h urinary toolkits were sent on the second day to the participants. The toolkits included a polypropylene bucket (4 liters, with a lid), a urine collection instruction, and two label stickers for writing names, dates, and ID. The instruction was to help the subjects collect urine correctly, including (1) starting the collection after the first urination in the morning, (2) collecting all the urine within 24 h, especially the first one of the next day, (3) keeping the container in the shade and avoiding contamination by blood or stools, and (4) keeping usual eating and drinking habits during collection.

Participants were required to submit their urine and dietary records on the fourth day. Research assistants checked urines by asking whether the subjects adhered to the collection steps and then observed the total volumes via naked eyes. The urinary buckets were transported to the laboratory for processing. The total volume of the sample was uniformly measured by research assistants using a measuring cup of 5 liters; the samples of more than 500 ml were considered valid. All the samples were then sub-packaged and sent to the clinical laboratory department within 2 h. Twenty-four-hour urinary sodium and potassium concentration was detected by ion-selective electrode method (Beijing LEADMAN Biochemical Co., Ltd., China) and converted into milligrams (one mmol/24 h U_Na_ = 23 mg/24 h sodium, one mmol/24 h U_K_ = 39 mg/24 h potassium).

### 2.5. Covariates

Information on the characteristics and eating habits of the subjects was collected along with the dietary surveys through online questionnaires. Characteristics data includes students’ age, sex, ethnicity, grade, major, and monthly pocket money. Eating habits, main ways of eating, and the frequency (amount) of re-adding salt to cooked meals were collected by self-designed questions.

### 2.6. Statistical analyses

EpiData 3.1 software (The Epi Data Association, Odense, Denmark), the Nutrition Star Expert System software (Zhending Health Technology Co., LTD., Shanghai, China), and Microsoft Excel software were employed to create the dietary database. IBM SPSS 26.0 (IBM, Washington, United States.) was used for data analyses. The figures were presented by R and MedCalc (Version 20.027, MedCalc Software, Ostend, Belgium). The Shapiro–Wilk test was used to identify the data distribution, and the non-parametric methods were employed for further analysis. Values are described as mean with standard deviation or median with quartiles. The *Spearman* correlation and intra-class coefficients (*ICC*) were calculated for the reproducibility between the test and retest results. The correlation coefficients <0.5 represent poor reliability, between 0.5 and 0.75 as moderate reliability, 0.75 and 0.9 as good reliability, and > 0.9 as excellent reliability (27). For validity, both 24 h U_Na_ and 3-day food records were tested against the results from Sodium-FFQ 1.0 via the following analyses (24, 28): (1) *Spearman* correlation coefficient to evaluate the correlation between Sodium-FFQ and other methods, (2) de-attenuated correlation coefficient to correct the observed correlation for the attenuating effect of random within-person error, (3) receiver operating characteristic analyses to identify the cut-off values, (4) *Bland–Altman* method and cross-classification proportion to assess the agreement of bias. According to Lombard (28), correlation coefficients >0.5 were considered good outcomes, between 0.2 and 0.49 as acceptable outcomes, and < 0.2 as poor outcomes. For the *Bland–Altman* analyses (12), the presence of bias, direction, and extent was described; the absence of significant correlations between the differences and the means of the tools was considered a good outcome. For the cross-classification, the agreement of ≥50% in the same category and ≤ 10% in the opposite is acceptable (12). Significant levels were set at *p* < 0.05.

## 3. Results

### 3.1. Characteristics of participants in the validation stage

A total of 102 college students were recruited into the study; 10 did not return their diet records and urinary sample, and 11 had abnormal urines. Eighty-one college students finished the FFQ survey and 24-h urine collection. The average age of the participants was 20.86 ± 6.51 years old (Table 1), including 21 males (25.9%) and 60 females (74.1%). Among them, 88.9% of college students are of Han nationality, and 55.6% have lived in Hunan Province for less than 3 years. There were 28 students (34.6%) from senior grades or above, and most (71.6%) were from medical majors. More than half of college students eat in student canteens (59.3%), followed by take-out (24.7%). Fifteen college students (18.5%) re-added salt to their cooked meals. There was no difference between the sex in most characteristics.

**Table 1:** Characteristics of participants in validation study (*n* = 81, % or *mean* ± *SD*).

Variables	Total 81 (100.0)	Sex	
		Boys 21 (25.9)	Girls 60 (74.1)
Age	20.86 ± 6.51	23.10 ± 12.61	20.08 ± 1.14
*Ethnicity*			
Han	72 (88.9)	19 (90.5)	53 (88.3)
Others	9 (11.1)	2 (9.5)	7 (11.7)
*Self-reported BMI (kg/m^2^)*			
< 18.5	13 (15.9)	2 (9.5)	11 (18.0)
18.5 ~ 23.9	55 (67.1)	14 (66.7)	41 (67.2)
24.0 ~ 27.9	11 (13.4)	4 (19.0)	7 (11.5)
≥ 28.0	3 (3.7)	1 (4.8)	2 (3.3)
*Grade*			
Freshman	12 (14.8)	4 (19.0)	8 (13.3)
Sophomore	17 (21.0)	3 (14.3)	14 (23.3)
Junior	24 (29.6)	7 (33.3)	17 (28.3)
Senior or older	28 (34.6)	7 (33.3)	21 (35.0)
*Major*			
Humanities & Social Sciences	7 (8.6)	2 (9.5)	5 (8.3)
Literature/Sciences and Engineering/Agriculture	16 (19.8)	7 (33.3)	9 (15.0)
Medicine	58 (71.6)	12 (57.1)	47 (76.7)
*Pocket Money (RMB, yuan / monthly)*			
< 1,000	11 (13.6)	2 (9.5)	9 (15.0)
1,001 ~ 1,500	25 (30.9)	7 (33.3)	18 (30.0)
1,501 ~ 2000	31 (38.3)	12 (57.1)	19 (31.7)
> 2000	14 (17.3)	0 (0)	14 (23.3)
*Main ways of eating*			
Student canteen	48 (59.3)	11 (52.4)	37 (61.7)
Take-out eating	20 (24.7)	5 (23.8)	15 (25.0)
Restaurant	11 (13.6)	5 (23.8)	6 (10.0)
Home	2 (2.5)	0 (0)	2 (3.3)
*Re-adding salt to cooked meals*			
Yes	15 (18.5)	5 (23.8)	10 (16.7)
No	66 (81.5)	16 (76.2)	51 (83.3)

BMI, Body mass index; RMB, renminbi; Chinese official coupons, 1 RMB ≈ 0.15 USD.

According to 24 h urinary sodium excretion, dietary sodium intake was estimated to be 2998.72 ± 1338.31 mg/d, with a median of 2664.55 (2011.93, 3901.95) mg/d. The dietary sodium intake of 81 college students from 3-day diet records was 1200.47 ± 575.84 mg/d, with a median of 1097.27 (784.87, 1425.70) mg/d. Since no college students cooked alone, the subjects could not record salt and most high-sodium seasonings in the 3 × 24 h dietary records. There was a difference between the 3 × 24 h dietary record and the sodium intake estimated by 24 h urinary sodium. The average urinary potassium was 992.43 ± 388.23 mg/d, and the median was 991.18 (671.78, 1265.26) mg/d. The median of 24 h urinary sodium to potassium ratios in the 81 participants was 5.52 (3.59, 7.38).

### 3.2. Reproducibility

The sodium intake, regardless of adjusting by re-adding salt, was significantly higher in the first FFQ survey than in the second (Table 2, *Wilcoxon U* = −2.856, *p* = 0.004). The intra-class correlation (*ICC*) between the test and retest results of Sodium-FFQ and the adjusted one was 0.571 (*p* < 0.001) and 0.575 (*p* < 0.001), respectively. The *ICC* of the food categories ranged from snacks of 0.619 (*p* < 0.001) to seasonings and others of 0.820 (*p* < 0.001), while the associations of staples, beans and products, meat and aquatic, eggs, and beverages were not observed.

**Table 2:** The sodium intake from FFQ in the test–retest investigation, mg/day (*n* = 81, *Median*, *P_25_* ~ *P_75_*).

Variables	Test	Retest	*ICC* (*p*-value)
FFQ	1509.58 (699.67, 2406.56)	1040.44 (502.17, 1929.88)	0.571 (< 0.001)
FFQ-adj	1566.18 (699.67, 2506.56)	1078.89 (566.91, 1964.92)	0.575 (< 0.001)
Staple food	41.06 (0.00, 100.35)	34.06 (0.00, 79.02)	0.230 (0.122)
Beans and products	0.34 (0.00, 20.27)	0.00 (0.00, 4.29)	0.254 (0.097)
Vegetable & Fungi	38.94 (18.66, 81.01)	35.41 (11.46, 73.09)	0.639 (< 0.001)
Fruits	0.10 (0.00, 0.27)	0.00 (0.00, 0.14)	0.750 (< 0.001)
Dairy and products	61.08 (29.89, 157.61)	39.86 (6.64, 93.00)	0.626 (< 0.001)
Meat & Aquatic	152.36 (79.89, 245.02)	86.78 (33.95, 171.59)	−0.104 (0.671)
Eggs	0.00 (0.00, 0.00)	0.00 (0.00, 0.00)	0.217 (0.138)
Snacks	220.30 (113.10, 358.15)	185.06 (68.76, 365.23)	0.619 (< 0.001)
Fast food	380.44 (116.63, 1163.84)	230.25 (20.22, 1100.66)	0.773 (< 0.001)
Beverages	0.00 (0.00, 1.79)	0.00 (0.00, 1.79)	0.276 (0.076)
Seasonings & Others	52.88 (0.00, 165.26)	26.44 (0.00, 86.32)	0.820 (< 0.001)

FFQ, the Sodium-FFQ; FFQ-adj, adjusted by salt re-added in cooked meals.

The *Spearman* correlation coefficients between the test–retest results on Sodium-FFQ were 0.654 (*p* < 0.001) and 0.672 (*p* < 0.001) on adjusted results. The *Spearman* coefficients ranged from 0.273 (*p* < 0.05) for staple foods to 0.678 (*p* < 0.05) for fast food. The food categories, except meat & aquatic, and eggs, presented moderate reproducibility in the test–retest investigation.

### 3.3. Validity

The *Spearman* coefficients of 3-day dietary records with Sodium-FFQ and the adjusted one were 0.393 (*p* < 0.05) and 0.409 (*p* < 0.01), respectively (Table 3); the *Spearman* correlation coefficients between them with 24 h U_Na_ were 0.342 (*p* < 0.01) and 0.329 (*p* = 0.004), respectively. Compared with the unadjusted FFQ, the adjusted one performed poorly, correlating with 24 h U_Na_. After adjustment using 24 h U_Cr_ and U_K_, the *Spearman* partial correlation coefficients between the Sodium-FFQ, adjusted one and 24 h U_Na_ were both 0.352 (*p* = 0.002); that of the Sodium-FFQ, adjusted one with 24 h sodium-to-potassium ratio were 0.370 (*p* = 0.001) and 0.358 (*p* = 0.001), respectively.

**Table 3:** *Spearman* Correlation coefficients between the Sodium-FFQ, food records, and 24 h U_Na_ (*n* = 81).

Variables	FFQ		FFQ-adj	
	Crude	De-attenuated	Crude	De-attenuated
3-day dietary record	0.393 (< 0.001)	0.485	0.409 (< 0.001)	0.459
24 h U_Na_	0.342 (0.003)	0.407	0.329 (0.004)	0.351
24 h U_Na_ (Adjust: 24 h U_Cr_)	0.319 (0.005)	0.379	0.318 (0.005)	0.339
24 h U_Na_ (Adjust: 24 h U_Cr_ + U_K_)	0.352 (0.002)	0.419	0.352 (0.002)	0.376
Na/K ratio	0.370 (0.001)	0.440	0.358 (0.001)	0.382

FFQ-adj, adjusted by salt re-added in cooked meals. Crude: crude Spearman correlation coefficients with *p*-value. Deattenuated: de-attenuated correlation coefficients: correcting coefficients referring to Liu. and Beaton., et al. (10). Deviation of 3 day dietary record, within-person, $s_{w_{a}}^{2}$ = 3668.568^2^. Between person, $s_{b_{a}}^{2}$ = 6493.668^2^, $\lambda_{a}$ = 0.319.

Deviation of test and retest results, within-person, $s_{w_{b}}^{2}$ = 6879.920^2^. Between person, $s_{b_{b}}^{2}$ = 12959.571^2^, $\lambda_{b}$ = 0.282.

Deviation of adjusted test–retest results, within-person, c: $s_{w_{c}}^{2}$ = 6879.920^2^. Between person, $s_{b_{c}}^{2}$ = 13044.052^2^, $\lambda_{c}$ = 0.278.

De-attenuated *Spearman* correlation coefficients presented the corrected correlation between Sodium-FFQ with the calibrated indices. The correlation was all expanded after correcting, with about 1.234 folds between Sodium-FFQ with 3-day dietary records and 1.190 folds with urinary indices.

**Figure 2:**
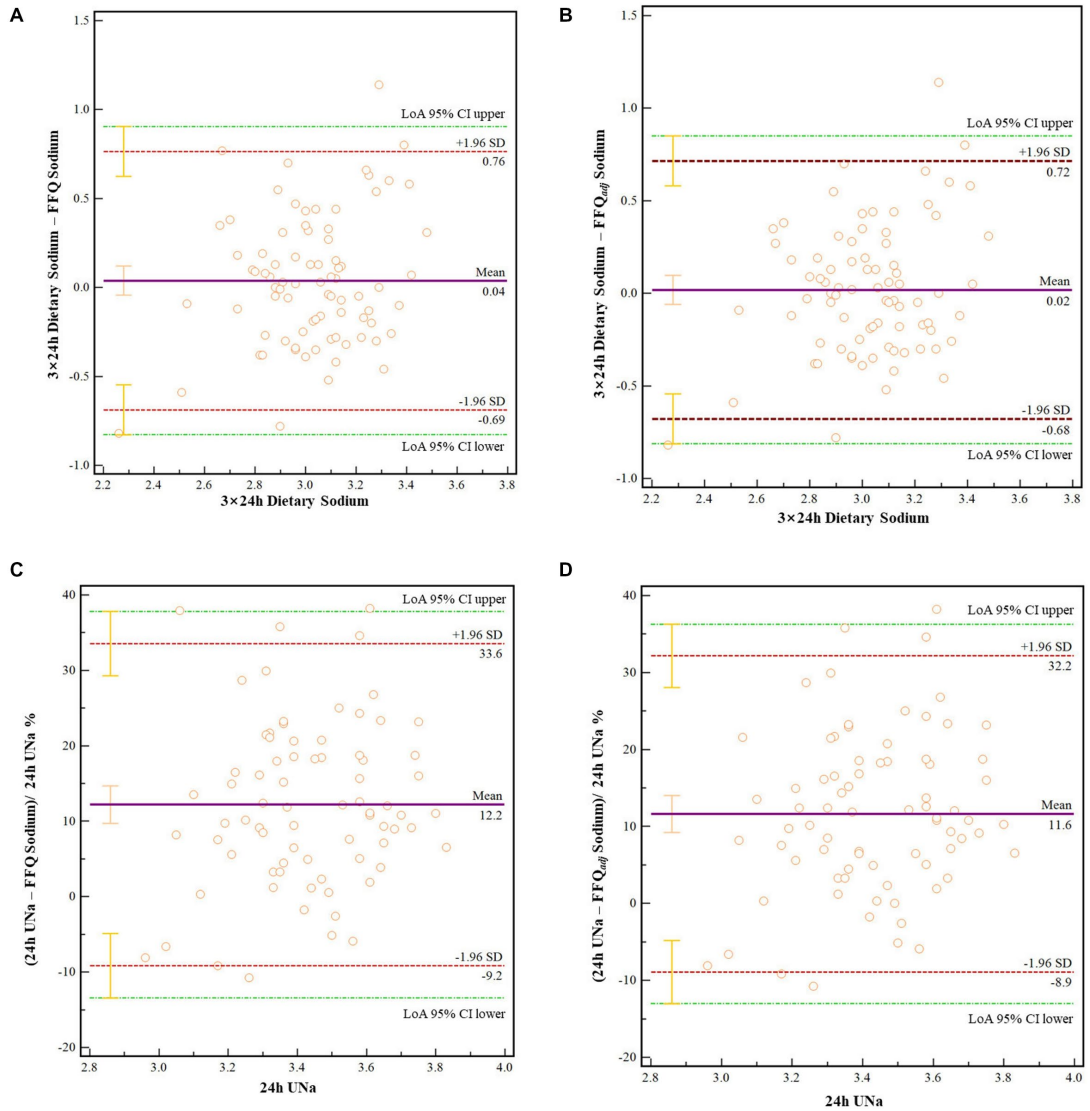
**(A)** *Bland–Altman* for the Sodium-FFQ and 3-day dietary record. **(B)** *Bland–Altman* for the adjusted FFQ and 3-day dietary record. **(C)** *Bland–Altman* for the Sodium-FFQ and 24 h U_Na_. **(D)** *Bland–Altman* for the adjusted FFQ and 24 h U_Na_. FFQ-adj, adjusted by salt re-added in cooked meals. LoA 95% CI upper/lower: Upper or lower of the 95% Confidence interval of the limits of agreements.

In order to assess the agreement, the presence, and the direction of bias at a group level, *Bland–Altman* analyses were used (Figure 2). For the FFQ, the mean bias with the reference measures was 0.04 g (Figure 2A, 95% CI: − 0.043, 0.121, *p* = 0.351) for the 3 day food record and 12.2 g (Figure 2C, 95% CI: 9.706, 14.687, *p* < 0.001) for the 24 h U_Na_. The FFQ-adj presented a mean bias of 0.02 g with the 3 day food record (Figures 2B) and 11.6 g with the 24 h U_Na_ (Figures 2D).

### 3.4. Agreement of the sodium-FFQ and 24 h U_Na_

#### 3.4.1. Roc analyses for the Cut-Off value

**Figure 3:**
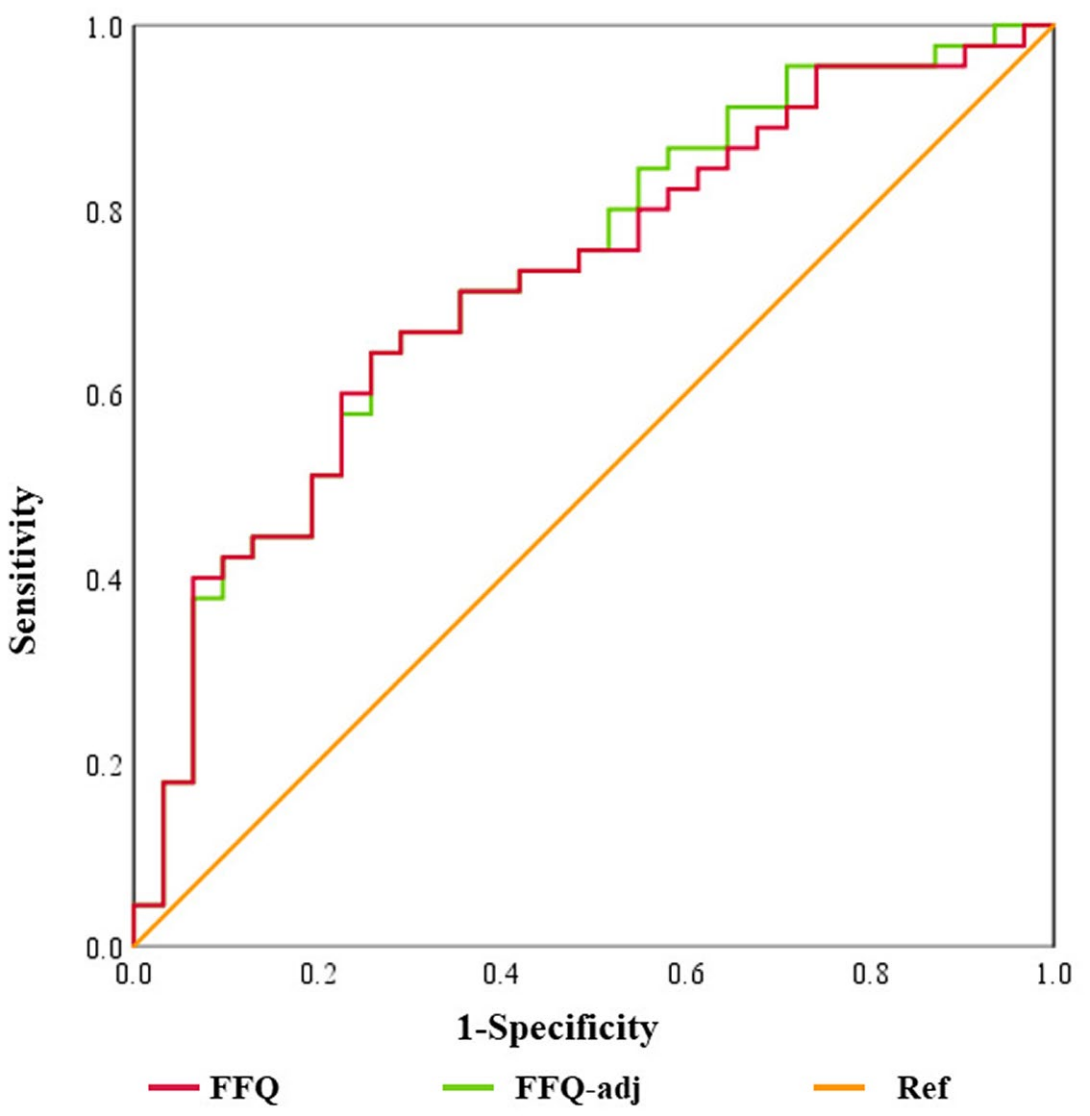
ROC curves of the Sodium-FFQ and the adjusted one. FFQ, the Sodium-FFQ. FFQ-adj, adjusted by salt re-added in cooked meals. *Ref*: diagonal line.

According to the sodium intake estimated by 24 h urine, participants were divided into “normal intake” and “high intake” groups, referred to the threshold of 2300.0 mg/d (2). ROC curves were drawn to identify the cut-off value of the Sodium-FFQ (Table 4; Figure 3). The area under the curve (AUC) of Sodium-FFQ was 0.728 (*p* = 0.001), with the threshold for classification of 1078.89 mg/d. The threshold of the adjusted one was 1150.92 mg/d (AUC = 0.719, *p* = 0.001). The two curves had no statistical difference in AUC (*p* = 0.292).

**Table 4:** ROC analyses of the Sodium-FFQ and adjusted one for cut-off values.

Variables	AUC	*p*	Youden-index	sensitivity	specificity	cut-off value
FFQ	0.728	< 0.001	0.386	64.44%	74.19%	> 1078.89
FFQ-adj	0.719	< 0.001	0.386	64.44%	74.19%	> 1150.92

#### 3.4.2. *Kappa* coefficients of the two methods

A cross-classification analysis was applied to evaluate the agreement at the individual level by cut-off values of 1078.89 mg/d and 1150.92 mg/d separately. The proportion of agreement was 68.4%, and that in the opposite group was 10.5%. The *Kappa* coefficients were both 0.371 (*p* = 0.001) of the Sodium-FFQ and the adjusted one.

## 4. Discussion

This study aimed to develop an assessment tool on dietary sodium intake (Sodium-FFQ) and present the measurement properties among college students. The Sodium-FFQ included the high-sodium foods consumed by undergraduates outside the campus cafeterias. Results showed acceptable reproducibility, validity, and agreement on the classification of the Sodium-FFQ. This Sodium-FFQ might be a helpful tool to assess dietary sodium intake and identify the primary sources for undergraduates. Further, it could also act as an alert to excessive sodium, contributing to sodium reduction in China.

In this study, the Sodium-FFQ is little different from sodium-related FFQ published yet (12, 15, 29). College students, who hardly cook themselves, are the target population of the Sodium-FFQ. Neither can they accurately estimate the amount of salt and high-sodium seasonings added by cooks nor restrict the intake of these seasonings. Hence, natural, processed, and fast foods, rather than salt and most high-sodium seasonings, are the body of the Sodium-FFQ. Additionally, Sodium-FFQ contained the foods in different seasons since food intake varies with time (30). Some foods rich in potassium (K > 150 mg/100 g), such as fresh vegetables and fruits, were also added to the food list due to the relationship between dietary potassium intake and sodium excretion (31). Despite being different from the previous FFQ, it is still available to assess sodium intake for college students.

The results showed significant differences among 24 h U_Na_, 3-day dietary records, and the Sodium-FFQ. The possible explanation might be that the 3-day dietary records and FFQ survey did not ask about the students’ salt and high-sodium seasonings intake, while 24 h urine contained total sodium intake daily. This could also be reflected by the results of *Bland–Altman* analyses, that the Sodium-FFQ had a good consistency with the dietary recording method but poorly with the 24 h U_Na_ method. Moreover, the difference between 24 h U_Na_ and dietary records were consistently observed in previous studies (12, 15, 29). The extensive distribution of sodium in natural foods, variation in the proportion of salt retained in food, the widespread utility of sodium compounds in food process and medication, as well as uncommon foods unlisted in food composition tables, could contribute to such a difference, too (25, 32, 33).

Our study found that sodium intake measured in the retest survey was lower than in the test survey, regardless of whether the Sodium-FFQ was adjusted. This is similar to most FFQ reproducibility research (15, 17, 24, 34). The training effect could explain it partially (30). Repeated measurement would make subjects sensitive to dietary intake and cautious about answering FFQ in the retest survey (30). Besides, the list of food weights in the toolkit was gifted; it might help some participants learn to estimate portion sizes between the two surveys. Other possible reasons are the Hawthorne effect (35) and volunteer bias (36).

Reproducibility presented the repeatability of the FFQ evaluated at two different time points (10). The current study demonstrated an acceptable reproducibility of the Sodium-FFQ, consistent with those of other studies (12, 34). The intra-group correlation coefficient (*ICC*) also revealed a moderate association between the test and retest of 0.571, within the range of previous studies from 0.43 to 0.97 (33, 34, 37). The *ICC* varies significantly among studies might be because of the test–retest interval (25), sample size (10, 24), dietary disparities (38), and sex and age differences of subjects (13, 33). In our study, taking the day-to-day variation of sodium intake and excretion (39, 40), as well as the possibility of high compliance (10), 2 weeks were chosen (12, 30, 34) to represent the short-term stability of it.

Consistency with the previous works (14, 15, 17, 24, 25, 33, 34, 37, 41), the correlation coefficient between the Sodium-FFQ and 3-day dietary records were 0.393 (*Spearman*) and 0.485 (de-attenuated), respectively. The correlation coefficients between Sodium-FFQ and 24 h U_Na_ were lower than Sodium-FFQ with dietary records; this was in line with the existing studies (ranging from 0 to 0.37) (15, 24, 25). However, after adjusting for 24 h U_Cr_ and 24 h U_K_, the partial correlation coefficient increased to 0.352 (*Spearman*) and 0.419 (de-attenuated), respectively. Such a weak correlation between 24 h U_Na_ and the Sodium-FFQ could be explained by the temperature effect on sodium excretion (42), single 24-h urine collection (24, 25), and relatively small sample sizes (10, 25). Nonetheless, these results presented an acceptable performance (both reproducibility and validity) of the Sodium-FFQ.

One notable result of our study is the association between Sodium-FFQ and 24 h urinary sodium-to-potassium ratio. Previous studies revealed a positive association between 24 h urinary Na/K ratio and blood pressure (40, 43). It has recently been reported to be a better predictive index for cardiovascular risk than urinary sodium or potassium excretion alone (40, 43, 44). Though the correlation performed poorly, it reveals the possibility of using Sodium-FFQ for college students’ diet guidance.

According to the criteria, the Sodium-FFQ presented a moderate reproducibility with 0.571 of *ICC*, and acceptable validity, with a series of *Spearman* correlation coefficients within 0.2 ~ 0.49, 68.4% being classified in the same category and 10.5% to opposite ones, and non-significant *p*-value in Bland–Altman test. Though the Sodium-FFQ did not meet the criteria of tools being applied to nutritional epidemiological studies (12), the results confirmed that foods outside canteens have become dominant among college students. These also hinted that timely approaches to establishing a low-sodium diet are necessary. Furthermore, the ultimate purpose of our study was to develop a screening tool that makes it possible to classify individuals according to intake levels, raise public awareness of the low-sodium diet, and promote healthier eating habits in college students. Therefore, we hope this study will help not only the practitioners of salt reduction but also college students and their parents.

This is the first study focused on developing and validating the food frequency questionnaire targeting college students’ sodium intake in China, which provides information and helps to salt reduction initiatives. The primary strength of the present study is that the tool was developed for a specific-vulnerable population (college students), who could contribute a lot to themselves and surrounding people. Another advantage is that two reference methods were used to test the validation of this Sodium-FFQ. Rigorous quality control procedures were carried out throughout the study. All the urine samples were examined, measured, and sub-packaged by uniformly trained assistants, guaranteeing the reliability of biochemical information. Additionally, each food item of the Sodium-FFQ was accompanied by pictures with portion size; this could help the respondents fill it more accurately.

Our study also had several limitations. First, a single 24-h urine collection could not fully represent an individual’s dietary sodium intake since it is associated with intra-individual variability under a controlled environment, furtherly contributing to the limitations. Furthermore, we collected the anthropometric information via a questionnaire instead of measurement by ourselves. College students, who are sensitive to body shape and dietary intake, might underreport their daily dietary consumption, BMI, and other actual situations; these could result in a lower intake from dietary records than actual ones. Moreover, the performance of the Sodium-FFQ was susceptible to relatively small sample sizes due to twenty-one participants’ drop-out. In addition, the students were convenience sampled from four comprehensive universities in the capital city of Hunan Province in central China. Hence, the food items in Sodium-FFQ could not be extrapolated to students all over China due to dietary disparities and the non-probability sampling methods.

## 5. Conclusion

A food frequency questionnaire targeting dietary sodium intake among college students has been developed and validated. The questionnaire includes forty-eight items from 11 food groups, demonstrating acceptable reproducibility and validity against the gold standard indicator of 24 h U_Na_, and a moderate classification agreement with the cut-off value of 1078.89 mg/d. Sodium-FFQ would be a potential tool to address exceedingly sodium intake from foods outside student canteens among college students. Further study should focus on modifying and re-validating the Sodium-FFQ for extrapolation. Simultaneously, it is necessary to develop Sodium-FFQ for people in different life stages and combine it with various platforms or electronic devices to contribute to sodium reduction.

## Data availability statement

The raw data supporting the conclusions of this article will be made available by the authors, without undue reservation.

## Ethics statement

The studies involving human participants were reviewed and approved by Ethics Review Committee of the Xiangya School of Public Health, Central South University. The participants provided their written informed consent to participate in this study.

## Author contributions

YX, YL, and QL made concepts, designed the study, acquired funding, and managed data preparation. YX, LY, JC, JD, and YQ recruited participants. YX, CX, CY, JL, JH, HZ, MW, YP, and QX conducted the investigation, analyzed, and interpreted data. YX drafted the original manuscript. QL and YL reviewed and edited the manuscript. All authors interpreted the results, made a substantial contribution to the improvement of the manuscript, read and agreed to the published version of the manuscript.

## Funding

This study was supported by the Fundamental Research Funds for the Central Universities of Central South University [Grant no 2020zzts815] to YX and QL and Huxiang Youth Talent Support Program [Grant no 2020RC3063] to YL. The funders had no role in the study’s design, in the collection, analyses, or interpretation of data, in the writing of the manuscript, or in the decision to publish the results.

## Acknowledgments

We want to thank the teachers and students from Xiangya School of Public Health, Central South University, in Changsha, China, for their involvement in this investigation. We would also appreciate the college students from four comprehensive universities participation. We also thank the Department of Health Management and Department of Clinical Lab, The Third Xiangya Hospital, Central South University, for their support.

## Conflict of interest

The authors declare that the research was conducted in the absence of any commercial or financial relationships that could be constructed as a potential conflict of interest.

## Publisher’s note

All claims expressed in this article are solely those of the authors and do not necessarily represent those of their affiliated organizations, or those of the publisher, the editors and the reviewers. Any product that may be evaluated in this article, or claim that may be made by its manufacturer, is not guaranteed or endorsed by the publisher.

## Supplementary material

The Supplementary Material for this article can be found online at: https://www.frontiersin.org/articles/10.3389/fnut.2023.1062845/full#supplementary-material
