# Supplementary material for: Be aware of the sodium intake outside student canteens: development and validation of a sodium food frequency questionnaire in Chinese undergraduates
Source: Front Nutr. 2023 Jun 8;10:1062845. doi: 10.3389/fnut.2023.1062845 (PMC10285065; doi:10.3389/fnut.2023.1062845)
Supplement: Supplementary file 1 [file Data_Sheet_1.docx]

**Sodium Food Frequency Questionnaire
for Chinese Undergraduates**

For each food listed, fill in the box indicating how often, and the amount, on average, you have used during the past month. For each item, please circle the specified food representing your average intake and indicate the way of it (in the last column.) Follows are samples for reference.

**Sample：**

| FOODS | | Frequency of Intake | | | | Amounts | Where to eat |
| --- | --- | --- | --- | --- | --- | --- | --- |
|  |  | 0 ~ 1 per month | ____ times per month | ____ times  per week | ____ times per day |  |  |
| F1 | **Deep-Fried Dough Stick** | ***√*** |  |  |  |  |  |
| F3 | **Stuffed food**  (Steamed bun/dumplings/wonton/Shumai) |  |  | ***3*** |  | 1 punch | Take-out |
| F9 | **Celery** |  | ***2*** |  |  | 1 scoop | Canteen |

**Now, please fill in the questionnaire according to your own situation.**

| FOODS | | Frequency of Intake | | | | Amounts | Where to eat |
| --- | --- | --- | --- | --- | --- | --- | --- |
|  |  | 0 ~ 1 per month | ____ times per month | ____ times  per week | 0 ~ 1 per month |  |  |
| Staple Food | | | | | | |  |
| F1 | **Deep-Fried Dough Stick** |  |  |  |  |  |  |
| F2 | **Oil cake** |  |  |  |  |  |  |
| F3 | **Stuffed food** (Steamed bun/dumplings/wonton/Shumai) |  |  |  |  |  |  |
| Beans & Products | | | | | | |  |
| F4 | **Dried bean curd** |  |  |  |  |  |  |
| F5 | **Mung beans** (Mung bean porridge / Mung bean smoothie) |  |  |  |  |  |  |
| Vegetable & Fungi | | | | | | |  |
| F6 | **Beans** |  |  |  |  |  |  |
| F7 | **Bitter gourd** |  |  |  |  |  |  |
| F8 | **Carrot** |  |  |  |  |  |  |
| F9 | **Celery** |  |  |  |  |  |  |
| F10 | **Broccoli** |  |  |  |  |  |  |
| F11 | **Salted vegetables** (Pickled / preserved vegetable / pickled Chinese cabbage / dry pickled mustard) |  |  |  |  |  |  |
| F12 | **Laver** |  |  |  |  |  |  |
| F13 | **Oyster Mushroom** |  |  |  |  |  |  |
| F14 | **Kelp** |  |  |  |  |  |  |
| Fruits | | | | | | |  |
| F15 | **Banana** |  |  |  |  |  |  |
| F16 | **Papaya** |  |  |  |  |  |  |
| Dairy & Products | | | | | | |  |
| F17 | **Milk** |  |  |  |  |  |  |
| F18 | **Milk powder** |  |  |  |  |  |  |
| F19 | **Dairy products** (dairy slice / cheese / cream) |  |  |  |  |  |  |
| Meat & Aquatic Products | | | | | | |  |
| F20 | **Pork meat** |  |  |  |  |  |  |
| F21 | **Beef** |  |  |  |  |  |  |
| F22 | **Spiced Beef** |  |  |  |  |  |  |
| F23 | **Preserved meat** |  |  |  |  |  |  |
| F24 | **Chinese sausage** |  |  |  |  |  |  |
| F25 | **Fresh-water fish** (carp / crucian carp / grass carp / perch) |  |  |  |  |  |  |
| F26 | **Shrimp and Crab** (prawn / shrimp / river crabs / hairy crabs) |  |  |  |  |  |  |
| F27 | **Smoked aquatic products** (squid slices / dried small shrimps) |  |  |  |  |  |  |
| F28 | **Roast duck** |  |  |  |  |  |  |
| F29 | **Other edible sausages** (Sausage / Ham / Luncheon-meat) |  |  |  |  |  |  |
| Eggs | | | | | | |  |
| F30 | **Salted duck egg** |  |  |  |  |  |  |
| F31 | **Century egg** |  |  |  |  |  |  |
| Snacks | | | | | | |  |
| F32 | **Bread** |  |  |  |  |  |  |
| F33 | **Cake** |  |  |  |  |  |  |
| F34 | **Biscuit** |  |  |  |  |  |  |
| F35 | **Potato chips** |  |  |  |  |  |  |
| F36 | **Hot dog** |  |  |  |  |  |  |
| F37 | **Spicy gluten / Spicy bean products** |  |  |  |  |  |  |
| F38 | **Peanut** |  |  |  |  |  |  |
| F39 | **Traditional festival food** (mooncake / rice dumplings / glue pudding) |  |  |  |  |  |  |
| Fast Food | | | | | | |  |
| F40 | **Instant noodle** |  |  |  |  |  |  |
| F41 | **Hamburger** |  |  |  |  |  |  |
| F42 | **Fried chicken** |  |  |  |  |  |  |
| F43 | **Sandwich** |  |  |  |  |  |  |
| F44 | **River snails rice noodle** |  |  |  |  |  |  |
| Beverages | | | | | | |  |
| F45 | **Coffee**  (Starbucks/Nestle/TASOGAREDE/illy, *etc.*) |  |  |  |  |  |  |
| Seasonings & Others | | | | | | |  |
| F46 | **Chili sauce** |  |  |  |  |  |  |
| F47 | **Sauerkraut fish** |  |  |  |  |  |  |
| F48 | **Hot pot sauce** |  |  |  |  |  |  |
